# Associated risk factors and prevalence of human papillomavirus infection among females visiting tertiary care hospital: a cross-sectional study from Nepal

**DOI:** 10.1515/med-2025-1327

**Published:** 2025-12-17

**Authors:** Tika Bahadur Thapa, Anit Lamichhane, Saroj Kunwar, Puspa Raj Khanal, Manisha Sapkota, Sujina Maharjan

**Affiliations:** Department of Molecular Laboratory, Sumeru City Hospital Pvt Ltd, Lalitpur, Nepal; Department of Pathology, Sumeru Hospital Pvt Ltd, Lalitpur, Nepal; Department of Laboratory Medicine, Manmohan Memorial Institute of Health Sciences, Kathmandu, Nepal

**Keywords:** human papillomavirus, cervical cancer, high-risk HPV, Nepal

## Abstract

**Objectives:**

There is a greater risk of cervical neoplasia in women with ongoing cervical infections with high-risk human papillomaviruses. This study aimed to determine the prevalence and associated risk factors for human papillomavirus infection among females visiting tertiary care hospital.

**Methods:**

This cross-sectional study was conducted over seven months with 376 study participants. Cervical swab samples were collected, and HPV types were detected using polymerase chain reaction. The associated risk factors for HPV infection were analyzed via binary logistic regression analysis.

**Results:**

The mean age of the female participants was 38.98 ± 8.74 years. The majority of the participants were females aged 30–39 years. The overall HPV prevalence was 15.7 % (59/376), 37 % of which were HPV-16 and 20 % of which were HPV-18. Logistic regression revealed that the likelihood of contracting HPV infection was greater among married females (AOR 4.576) and who did not used contraceptive (condoms) (AOR 1.559).

**Conclusions:**

Our findings highlighted the notable prevalence of high-risk HPV-16 and HPV-18 and emphasized the importance of public awareness regarding risk factors of HPV infection, along with a specific emphasis on vaccination, promoting testing and early detection methods to curb the progression of cervical cancer.

## Introduction

The human papillomavirus (HPV) is one of the most common sexually transmitted viruses. There are approximately 100 different HPV genotypes; approximately 40 are known to infect the vaginal tract. Eighteen types, in particular, HPV-16 and HPV-18, described as high-risk/oncogenic types (HR-HPV), and often associated with the development of cervical cancer [1]. In addition, cervical intraepithelial neoplasia is linked to persistent cervical infection with HR-HPV genotypes [2]. According to the World Health Organization (WHO), the incidence of cervical cancer is increasing among women in low- and middle-income countries (LMICs), with an estimated 88 % of 604,127 new cases annually. With 2,244 new cases and 1,493 fatalities annually, cervical cancer remains the most common malignancy among women overall in Nepal [3] and the second most common cancer in women between the ages of 15 and 44 years [4].

Previous studies have shown that oncogenic HPVs are the primary agents for developing cervical cancers [5]. Depending on its carcinogenic potential, HPV is classified into high-risk (HR) or low-risk (LR) types. Epidemiological studies have shown that HPV-16 and 18 account for more than majority of HR-HPV infections worldwide [6]. In Nepal, it is estimated that 2 % of women in the general population have cervical HPV-16/18 infection at any given time and that HPV-16 or 18 is responsible for 80.3 % of invasive cervical malignancies [4]. The ability of HR-HPV testing is being evaluated as a cancer screening method, and vaccines targeting HR-HPV may significantly reduce the burden of cervical cancer. Thus, the detection and control of HR-HPV genotypes have become the focus of cervical cancer prevention strategies.

Early sexual activity, sexual promiscuity, increased lifetime sexual partners, and poor hygienic environments, low public health awareness are the risk factors related to the increased prevalence of HPV infection. HPV is a common sexually transmitted infection, and almost all sexually active people are infected at some point in their lives. The appropriate use of contraceptive condoms can help prevent HPV infection but does not cover total protection [7].The secondary preventive approaches for cervical cancer include efficient population-based screening, Pap smear tests, HPV DNA testing, and HPV vaccines. These measures contribute to a high rate of successful reduction in HPV infection and subsequent development of cervical cancer [8].

More than 80 % of women living in rural areas of Nepal are at particular risk of delayed diagnosis of cervical cancer, and infection with carcinogenic HR-HPV has almost always been found to be associated with cervical cancer [9]. Therefore, the need to detect these HR-HPV genotypes and apply appropriate vaccines is crucial for their control. In the context of Nepal, very few studies have documented the prevalence and associated risk factors for HR-HPV (HPV-16/18), which might be the possible reason for the increased prevalence of cervical cancers in Nepal. Thus, our present study aimed to determine the prevalence of high-risk HPV-16/18, their distribution among different age groups and potential risk factors for their infection among females who visited our hospital.

## Materials and methods

### Study design and population

This hospital-based cross-sectional study was conducted over seven months, from August 2022 to February 2023. All female patients aged above 20 years who visited the gynecology department were included in the study. All the women enrolled in this study completed a self-administered questionnaire about their demographic data. The women were then interviewed by trained nursing staff (data collectors) with respect to their reproductive and sexual life variables. The survey was conducted through personal, one-on-one interviews in a private setting within the gynecology department to minimize disruptions from other participants.

### Exclusion criteria

Women who were pregnant or recently gave birth, those who were unable to give consent owing to physical or mental incapacity, those who currently had a cervical cancer diagnosis, and those who were receiving anticancer treatment were excluded from the study.

### Sample collection

Cervical cells from the endocervix and ectocervix were collected with a cervix brush by a gynecologist. After being inserted into the endocervical canal and rotated gently 180°, the brush containing the cellular material was placed in a vial containing preservative buffer solution. The samples were stored at −20 °C for no more than three days.

### HPV-DNA extraction and amplification

HPV DNA was extracted from each sample collected via a manual DNA extraction kit (SpinStar™ Virus Nucleic Acid Kit, ADT Biotech). The SpinStarr™ Viral Nucleic Acid Kit procedure comprises four steps (lyse, bind, wash, elute). **Lysis and binding**: A 50 µL proteinase K mixture with a 200 µL cervical sample in a microcentrifuge tube was mixed well; a 215 µL mixture of the lysis buffer SSVL-carrier RNA was added to the same microcentrifuge tube; the mixture was incubated at 65 °C for 10 min and briefly centrifuged; 280 µL of ethanol was added and vortexed for 15 s; the mixture was transferred to a column and centrifuged at 8,000 rpm for 1 min; and the collection tube containing the filtrate was discarded and placed in a new collection tube. **Washing:** The mixture was washed with 500 µL of SSW1 buffer 1 (8,000 rpm for 1 min), the filtrate was discarded, and the column was transferred to another clean collection tube. Similarly, the mixture was washed with 500 µL of SSW2 buffer 2 (8,000 rpm for 1 min), the supernatant was discarded, and the collection tube was reused. Finally, 500 µL of ethanol was added to wash (centrifuge at 8,000 rpm for 1 min), the filtrate was discarded, and the collection tube was reused (centrifuge at 13,300 rpm for 10 min). The column was transferred to a clean microcentrifuge tube, and the collection tube containing the remaining ethanol was discarded. **Elution:** 30–60 µL of SSE elution buffer was added to the center of the column membrane, which was subsequently incubated at 30 °C for 5 min. The mixture was subsequently centrifuged at 8,000 rpm for 1 min, and the extraction filtrate was collected in a microcentrifuge tube.

HPV DNA was amplified via an HPV nucleic acid amplification kit from Jiangsu Mole Bioscience Co., Ltd. (fluorescent probe-based real-time PCR assay). The primers and fluorescent probes were specifically designed for the L1 loci of the HPV genome, and the fluorescent probes were labeled with FAM, HEX/VIC, and ROX fluorescent dyes. By using polymerase chain reaction (PCR) with TaqMan fluorescent probes, the types of 16, 18, 26, 31, 33, 35, 39, 45, 51, 52, 53, 56, 58, 59, 66, 68, 73, and 82 HPV DNA from the cervical shedding cells were qualitatively detected, and those samples were identified as 16, 18 or other types. The primers and probe sequences for the internal control (IC) were derived from human HAPB gene sequences, and the probe was specifically labeled with the CY5 fluorescent dye. For each batch of samples, both positive and negative controls were included. The positive control comprises fragments of the HPV16, 18, and 31 L1 genes, along with the HAPB gene. Conversely, the negative control consisted of a fragment solely from the HAPB gene. Upon completion of the reaction, the Ct value in the CY5 channel for the negative control should be ≤30.00. Additionally, the Ct values in the other three channels should also be negative. Conversely, the positive control should exhibit positive Ct values in all four detected channels. These criteria must be concurrently met in a single test; failure to do so deems the PCR invalid, necessitating a reperformance of the procedure. The Ct values in the FAM channel (HPV16) ≤34.48, HEX/VIC channel (HPV18) ≤35.23 and ROX channel (HPV other types) ≤35.29 were considered positive for HPV-16, HPV-18, and HPV-others, respectively.

### Statistical analysis

IBM SPSS version 23.0 (IBM Corp., USA) was used for data analysis. Descriptive statistics, such as frequency and percentage, were carried out. HPV incidence was calculated as a percentage, and the chi-square test was used to test the associations of demographic variables with HPV infection outcomes. Risk factors associated with HPV* *infection were calculated as unadjusted or crude odds ratio (COR) and adjusted odds ratios (AORs) and 95 % confidence interval (CI) via logistic regression models. p-Value <0.05 were considered statistically significant.

## Results

### Demographic characteristics of the study participants

This hospital-based cross-sectional study included a total of 376 female patients with a mean age of 38.98 ± 8.74 years (ranging from 20 to 70 years). Most of the females were from the 30 to 39 years age group, followed by the 40–49 years age group. The majority of females were married (91.8 %), and 68.1 % had a university-level education. Fourteen percent of females were smokers, and only 10.9 % of females had a history of sexually transmitted disease (STD). The highest number of females (73.9 %) reported the use of contraceptive condoms. According to the results of the chi-square analysis, study variables such as marital status, educational level, smoking habits, STD history and contraceptive condom use were significantly associated with HPV infection (Table 1).

**Table 1:** Demographic characteristics of the study participants.

Characteristics	Total patients (n=376)	HPV-negative (n=317)	HPV-positive (n=59)	p-Value
	n (%)	n (%)	n (%)	
Age group				
20–29 years	49 (13.1)	35 (11.1)	14 (23.7)	0.106
30–39 years	146 (38.8)	127 (40.0)	19 (32.2)	
40–49 years	137 (36.4)	116 (36.6)	21 (35.6)	
50–59 years	37 (9.8)	33 (10.4)	4 (6.8)	
≥60 years	7 (1.9)	6 (1.9)	1 (1.7)	

**Marital status**				

Married	345 (91.8)	295 (93.1)	50 (84.7)	**0.033** ^**a**^
Unmarried/Single	31 (8.2)	22 (6.9)	9 (15.3)	

**Education status**				

Primary school	30 (8.0)	21 (6.6)	9 (15.3)	**0.000** ^**a**^
High school	47 (12.5)	37 (11.7)	10 (16.9)	
University education	256 (68.1)	232 (73.2)	24 (40.7)	
Illiterate/No formal education	43 (11.4)	27 (8.5)	16 (27.1)	

**Smoking habits**				

Yes	53 (14.1)	29 (9.1)	24 (40.7)	**0.000** ^**a**^
No	323 (85.9)	288 (90.9)	35 (59.3)	

**STD history**				

Yes	41 (10.9)	25 (7.9)	16 (27.1)	**0.000** ^**a**^
No	335 (89.1)	292 (92.1)	43 (72.9)	

**Contraceptive use (condoms)**				

Yes	278 (73.9)	246 (77.6)	32 (54.2)	**0.000** ^**a**^
No	98 (26.1)	71 (22.4)	27 (45.8)	

HPV, human papillomavirus; STD, sexually transmitted disease. ^a^p<0.05=significant (Chi-square). Bold values to indicate statistical significance.

### HPV prevalence and type distribution

**Figure 1:**
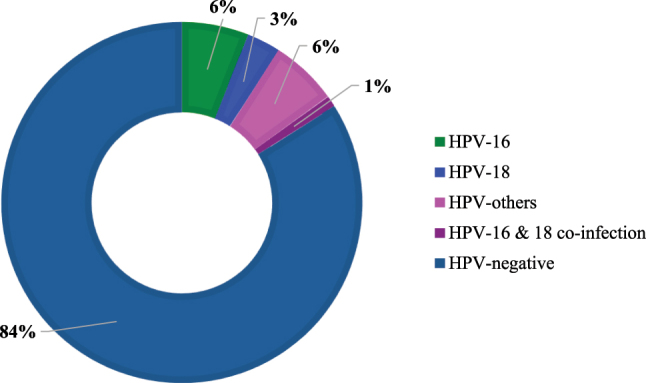
Distribution of HPV genotypes among the study participants.

The overall prevalence of HPV infection was 15.7 % (59/376) and remaining 84.3 % (317/376) were found to be HPV-negative. Among the positive cases, the majority of the HPV types were HPV-others (23/59), followed by HPV-16 (22/59) and HPV-18 (12/59). In two patients (2/59), coinfection of HPV-16 and HPV-18 was also found (Figure 1).

### Distribution of HPV infection among different age group

**Figure 2:**
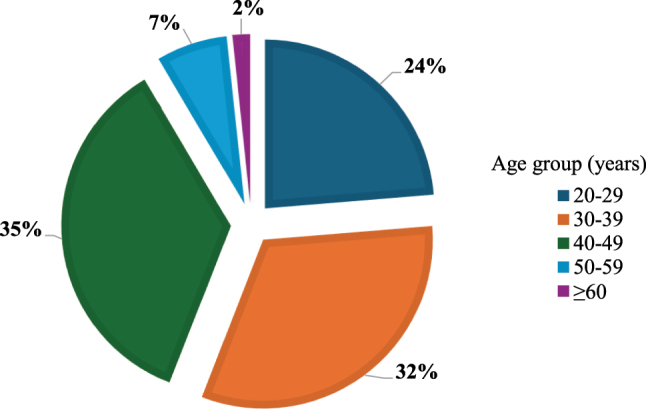
Overall distribution of HPV cases among different age groups*.*

**Figure 3:**
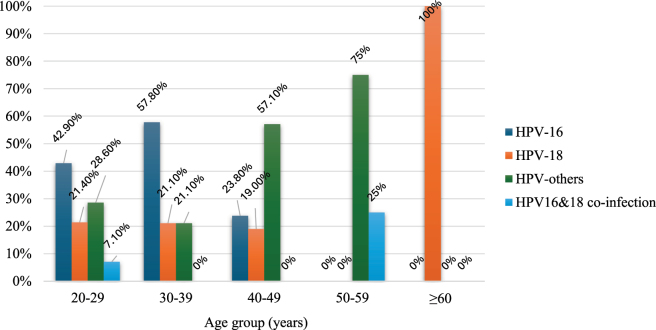
Percentage distribution of HPV genotypes among different age group of participants.

The distribution of HPV infection varied across different age groups, with most positive cases found among female participants aged 40–49 years (35 %) and 30–39 years (32 %). Then 27 % of positive cases were from age group 20–29 years and 7 % from 50-59 years of age (Figure 2). Among the age group 20–29 years, highest numbers of HPV genotype were HPV-16 followed by HPV-others. Similar pattern also observed in age group 30–39 years. However, in the age group 40–49 years and 50–59 years, HPV-others was in majority (Figure 3).

### Risk factors associated with HPV infection

Logistic regression was performed to identify independent risk factors associated with HPV infection (Table 2). In the univariate analysis, smoking (COR=0.147; 95 % CI: 0.077–0.280) and history of STDs (COR=0.230; 95 % CI: 0.114–0.465) were significantly associated with lower odds of HPV infection, while contraceptive use (condom) (COR=2.923; 95 % CI: 1.643–5.202) and marital status (COR=2.414; 95 % CI: 1.051–5.543) were associated with higher odds of HPV infection. In the multivariable model, after adjusting for potential confounders, smoking (AOR=0.167; 95 % CI: 0.082–0.338) and history of STDs (AOR=0.338; 95 % CI: 0.146–0.780) remained independently associated with lower odds of HPV infection, while marital status (AOR=4.576; 95 % CI: 1.873–11.181) remained significantly associated with higher odds. Contraceptive use (condom) did not retain statistical significance after adjustment (AOR=1.559; 95 % CI: 0.784–3.098).

**Table 2:** Binomial logistic regression analysis of factors associated with HPV infection (n=376).

Variables	Unadjusted analysis		Adjusted analysis	
	COR (95 % CI)	p-Value	AOR (95 % CI)	p-Value
Smoking habits				
Yes	0.147 (0.077–0.280)		0.167 (0.082–0.338)	
No	Reference	**0.000** ^**a**^	Reference	**0.000** ^**a**^

**STD history**				

Yes	0.230 (0.114–0.465)	**0.000** ^**a**^	0.338 (0.146–0.780)	**0.011** ^**a**^
No	Reference		Reference	

**Contraceptive use (condoms)**				

No	2.923 (1.643–5.202)	**0.00** ^**a**^	1.559 (0.784–3.098)	0.205
Yes	Reference		Reference	

**Marital status**				

Married	2.414 (1.051–5.543)	**0.038** ^**a**^	4.576 (1.873–11.181)	**0.001** ^**a**^
Unmarried	Reference		Reference	

STD, sexually transmitted disease; COR, crude odds ratio; AOR, adjusted odds ratio. ^a^p<0.05=significant. Bold values to indicate statistical significance.

## Discussion

The successful elimination of cervical cancer through vaccination and screening initiatives relies heavily on optimizing country-specific HPV data. Such information is critical for assessing the long-term effectiveness of HPV vaccines and HPV-based screening strategies, as well as for guiding policymakers in determining the most effective approaches for preventing and controlling cervical and other HPV-related cancers. Among HPV-related cancers, cervical cancers associated with HPV infections can be detected via HPV-DNA polymerase chain reaction. This led to the implementation of screening policies based on HPV-DNA PCR and the development of prophylactic vaccines [10], 11]. In developing countries such as Nepal, the use of HPV-DNA-based techniques for HPV detection is not yet widespread for diagnostic purposes. As a result, relying solely on cytological pap smear examination makes it challenging to identify the HPV genotypes [12]. Thus, understanding HPV genotype distribution is crucial for implementing effective vaccination programs, as previous studies have indicated that HPV genotypes can vary by geographical region and vaccination efforts should therefore focus on the prevalent genotypes in specific areas [13]. Thus, this study was aimed to determine the distribution of HPV genotypes among females visiting our hospital. We have found that 15.7 % (59/376) of the females who participated in our study were positive for HPV-DNA. The prevalence of high-risk HPV (HPV-16/18) was positive in 10 % (36/376) overall and 61 % (36/59) among the total positive cases. This result is similar to the earlier studies from Nepal in 2017–2018 by Shakya et al. and Thapa N et al. However, it is higher in comparison to the study which was conducted in 2010 from Nepal by Sherpa AT et al. The prevalence of HPV observed in our study higher than that reported from India (5.9 %) and Sri Lanka (6.2 %) but lower than the prevalence reported from Bangladesh (29.5 %) [14], [15], [16]. This comparison clearly confirms the notable prevalence of HPV in south Asian region and also showed the increasing trend of HPV cases over the period in Nepal.

The report from HPV Information Center Nepal has reported a link between high-risk HPV types and cervical cancer [4], highlighting the importance of HPV vaccines that target specific genotypes of HPV. The percentage of high-risk HPV cases was 7.9 % in the Shakya et al. and 11.9 % in the study by Thapa N et al., which is consistent in our study where 10 % of cases were high-risk HPV [17], [18], [19]. HPV prevalence varies by country; the World Health Organization has noted that developing countries have a higher HPV incidence than developed nations, which is attributed to lower rates of complete HPV vaccination [20]. According to the report of HPV information center Nepal 2023, it is estimated that 2 % of women in the general population have an HR-HPV (HPV-16/18) infection at any given time, and 80.3 % of invasive cervical cancer cases are due to HPV types 16 or 18 [4]. Similar results were also reported in studies performed in India, where the most common high-risk HPV were HPV-16 and HPV-18 [14], 21]. In contrast, studies from Thailand and Indonesia have shown that high-risk HPV types other than HPV-16 and 18 are more predominant [22], 23]. These comparisons revealed that there are differences in the prevalence of HPV genotypes among different countries and different geographical locations. Therefore, it is essential to conduct countrywide surveillance of HPV genotypes to identify the most prevalent HPV genotype and to design the suitable HPV vaccine tailored to the prevalent genotypes in specific populations.

The distribution of HPV is different among various age groups, and previous studies from Nepal reported that females between 20 and 40 years of age have the highest prevalence of HPV infections [17]. A very similar result was also observed in our present study, in which the majority of HPV-positive cases were from the 30–50 years age group, which is consistent with the results that has been reported from different parts of the world [21], 24]. This comparison indicates that sexually active females, particularly those of reproductive age are at higher risk of contracting HPV infection. However some studies have also reported a significant numbers of elderly women between 60 and 80 years of age with a greater prevalence of HPV infection [25], underscoring the widespread distribution of HPV among different age groups. Furthermore, the distribution of high-risk HPV revealed that most high-risk HPV16/18 strains were from the 20–40 years age group in our study. This result is consistent with the study by Babi A et al., who reported that most high-risk HPV-16/18 females were from 18-45 years of age [26]. This similarity suggested that the risk of high-risk HPV infection is greater among reproductive age group females and previous studies have reported a strong association between the sexual activity and the incidence of HPV infection [27]. Given the significant risk of developing precancerous lesions linked to high-risk HPV infections, it is advisable to conduct screening for cervical intraepithelial neoplasia (CIN) via cytological pap smears in the females of reproductive age group, and these results should be analyzed in correlation with HPV DNA testing [28], 29].

In this study, several sociodemographic and behavioral factors were associated with HPV infection, with non-use of contraceptive (condom) showing a higher prevalence of HPV compared with contraceptive (condom) users, consistent with longitudinal evidence that consistent contraceptive use reduces HPV acquisition and may promote viral clearance [30]. Although smoking and a history of sexually transmitted diseases (STDs) were more common among HPV-positive women in the descriptive analysis (Table 1), the logistic regression paradoxically indicated lower odds of HPV infection among smokers and those reporting history STDs. This finding diverges from the majority of prior studies, which consistently report smoking as a risk factor for HPV acquisition and persistence through local immunosuppression [31], 32] and STDs such as *Chlamydia trachomatis* as cofactors that facilitate HPV infection by disrupting epithelial barriers and altering the vaginal microenvironment [33], 34]. The inverse associations observed here in our study are most plausibly explained by methodological rather than biological factors, including confounding and multicollinearity factors like education status, and marital status, reporting bias and the cross-sectional design that captures prevalence rather than persistence, raising the possibility of reverse causality if individuals modified behaviors after prior diagnoses. Taken together, our data reinforce contraceptive use as a protective factor but suggest caution in interpreting the apparent protective effects of smoking and STD history; these likely reflect analytical limitations rather than true biological protection. Longitudinal studies with larger sample sizes, detailed behavioral and clinical covariates, and prospective follow-up are needed to clarify the temporal relationship between these exposures and HPV infection risk in this population.

The generalizability of our findings to the broader female population in Nepal may be limited. Nevertheless, our results highlight a substantial prevalence of high-risk HPV genotypes, particularly HPV-16 and HPV-18. Persistent infection with these high-risk types is a well-established risk factor for cervical intraepithelial neoplasia and invasive cervical cancer, conditions that can significantly impact reproductive health and fertility [35], 36]. Early detection of high-risk HPV infections through routine screening, combined with preventive strategies such as HPV vaccination, is critical to reducing the risk of progression to cervical cancer while preserving fertility potential [37]. For women diagnosed with HPV-related cervical cancer who may require fertility-sparing treatment, approaches such as oocyte vitrification following ovarian stimulation with antagonist protocols offer a viable means of safeguarding future reproductive capacity [38], 39]. Notably, evidence indicates that children born from vitrified oocytes or frozen embryos exhibit favorable neonatal outcomes and normal long-term development, underscoring the safety and feasibility of these fertility preservation strategies [40], 41]. In Nepal, the recent initiation of an HPV vaccination demonstration program, utilizing a bivalent vaccine targeting HPV-16 and HPV-18 in girls aged 10–14 years [42], represents an important preventive measure against HPV-related cervical neoplasia. However, it remains equally critical to focus on reproductive-aged women diagnosed with HPV-related cervical cancer, ensuring that fertility preservation is considered alongside oncologic management. Collectively, these results and comparisons underscore the need for integrated public health strategies that combine HPV prevention, early detection, and reproductive health preservation, thereby addressing both disease control and the maintenance of future fertility in women at risk.

## Conclusions

Our study identified a considerable prevalence of HPV infection among female patients attending our healthcare facility. Multivariate analysis revealed statistically significant associations between HPV positivity and demographic factors, including, marital status, and contraceptive practices. Notably, high-risk oncogenic HPV genotypes 16 and 18 were identified, underscoring their possible etiological relevance in cervical carcinogenesis. These findings recommend the critical need for routine HPV screening, implementation of prophylactic vaccination specifically targeting high-risk genotypes 16 and 18, and the development of effective early diagnostic interventions. Moreover, enhancing public health literacy regarding HPV transmission dynamics and associated oncogenic risks is imperative to promote adherence to preventive strategies and mitigate the burden of HPV-related diseases.
